# Pleomorphic adenoma of the soft palate: major tumor in a minor gland

**DOI:** 10.11604/pamj.2015.22.281.5227

**Published:** 2015-11-23

**Authors:** Mounir Hmidi, Hicham Aatifi, Ali Boukhari, Mohammed Zalagh, Abdelhamid Messary

**Affiliations:** 1 Department Otolaryngology and Neck Surgery, Military Hospital My Ismail Meknes, Morocco

**Keywords:** Pleomorphic adenoma, soft palate, salivary gland

## Abstract

Salivary gland tumors are a relatively rare and morphologically diverse group of lesions. Pleomorphic adenoma is a benign tumor of the salivary gland that consists of a combination of epithelial and mesenchymal elements. The tumor most commonly arises from the parotid (60-70%) or submandibular glands. It develops less frequently in a minor salivary gland, presenting as an intraoral mass depend on the soft palate. We describe a case of benign pleomorphic adenoma of soft palate in a 45 year old female with computed tomography and histopathological findings. This patient presented in ENT department with history of gradually increasing mass lesion in the palatal region over a period of few months.

## Introduction

Pleomorphic adenoma is the most common tumor (60%) of major and minor salivary glands, nearly 70% of the tumors of minor salivary glands are Pleomorphic adenomas, and the most common intraoral site is the palate, followed by upper lip and buccal mucosa [1]. Studies have reported that in world population 13.9-51.4% of all salivary gland tumors arise from an intraoral site and 34.7-67.1% of them are benign [2]. This is a tumor of diverse histological and topographical presentation. The differential diagnoses are many and atypical presentations of this tumor are not uncommon. We report a case of a pleomorphic adenoma with predominant plasmacytoid myoepithelial cells arising in minor salivary glands of the soft palate in a 45 year old female.

## Patient and observation

**Figure 1:**
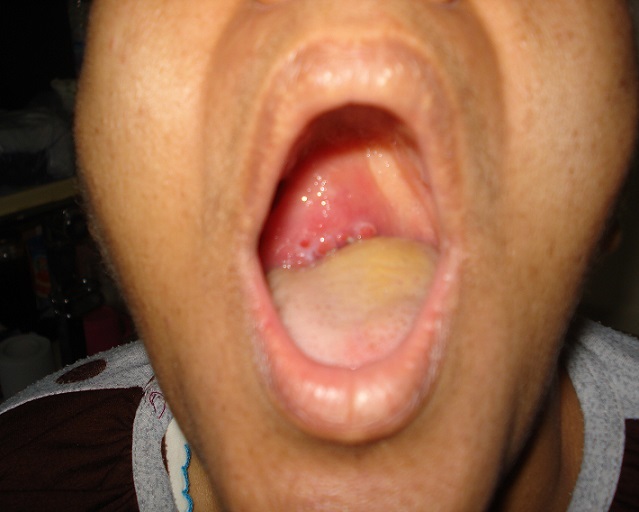
Lesion of mid part of palate, extending up to the posterior border of hard palate

**Figure 2:**
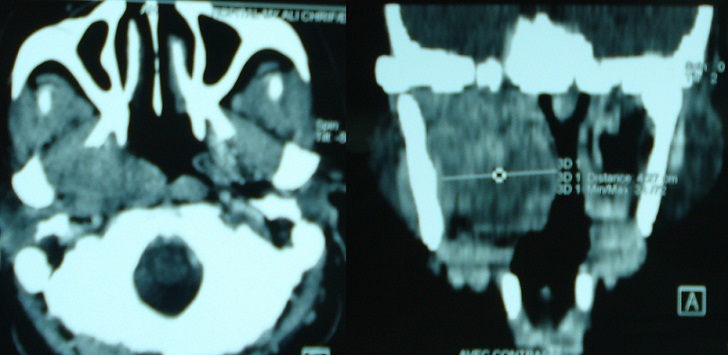
CECT axial and coronal planes demonstrating a well defined enhancing mass lesion arising from soft palate

**Figure 3:**
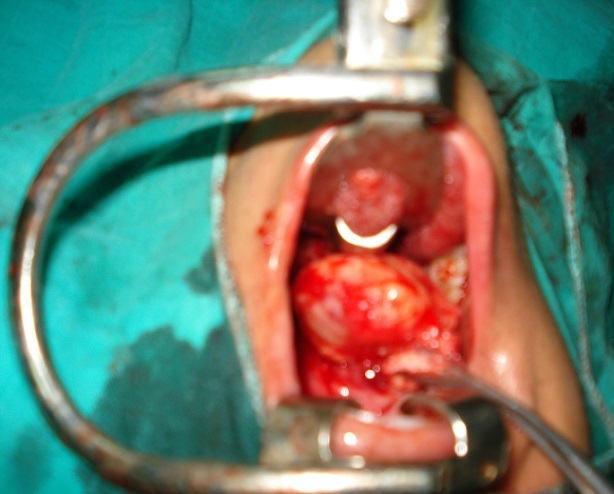
Surgical wound following tumor excision

A 45 year-old unmarried female, presented in the department of otorhinolaryngology with chief compliant of slow growing swelling over palate since last 6 months. The lady also complained of pain over swelling while swallowing along with difficulty in deglutition because of swelling. There was no history of headache, vertigo, ear discharge, trauma or any other associated symptoms. There was no history of fever, weight loss, bleeding, pus discharge or any other type of discharge from the swelling. The personal history of the patient did not reveal any history of smoking, or any other addiction. On past history there was no history of similar illness in past neither there was any past history of significant medical or surgical illness. There was no family history of similar complaints. The patient did not take any treatment for the above complaints and presented for the first time in our hospital for the similar complaints. General examination reveals normal and stable vital parameters. There was no icterus or pallor. The examination of remaining body did not reveal any other swelling. The systemic examination of other systems was within normal limits. On local examination there was a globular mass on right side of palate with normal overlying mucosa was seen. The lesion appeared to arise from mid part of palate, extending up to the posterior border of hard palate (Figure 1). It was non-tender, firm, and non compressible and had well defined margins. Posteriorly it was limited by the posterior margin of soft palate and anteriorly merging with the soft palate. Superiorly it was extending into nasopharyngeal region. The transillumination test was negative. Rest of otorhinolaryngological examination did not reveal any other significant abnormality. On the basis of history and clinical examination a diagnosis of solid non infectious mass of soft palate likely of benign etiology was made. The possibility of infective etiology (palatal abscess) was less likely as there no fever neither there was any tenderness or erythema. She was then advised a contrast enhanced computed tomography scan of paranasal sinuses. CT scan was performed on a dual source 128 slice spiral CT with coronal and saggital reformations. The CT scan revealed a well defined homogeneously enhancing hypodense soft tissue les ion of size 4,27 cm X 4cm X 4,5 cm arising from right side of soft palate and abutting the hard palate anteriorly, uvula posteriorly, tongue inferiorly and nasopharyngeal wall superiorly. Laterally it was seen to be abutting the right lateral nasopharyngeal wall (Figure 2). There was no infiltration of the surrounding structures with well defined surrounding planes. There was no non enhancing area within to suggest central necrosis of secondary infection. The radiological features were suggestive of a benign etiology neoplastic mass. The possibility of other clinical diagnosis of palatal abscess was completely ruled out. The patient was then advised surgical excision of the lesion. A biopsy was performed, which was suggestive of PA. All preoperative blood and urine investigations were done, which were within normal limits. The patient underwent surgery under general anesthesia. The excised mass was 5 × 4 cm (Figure 3) and surgical wound was closed with advancement of adjacent mucosa. Histopathological examination revealed epithelial and mesenchymal cells and along with other features was suggestive of pleomorphic adenoma. The patient′s postoperative course was uneventful. The healing was satisfactory. No recurrence was observed after a follow-up period of 1 year.

## Discussion

Pleomorphic adenoma is the most common tumor of major salivary glands, although approximately 80% of these are found in parotid gland. About 4-5% of pleomorphic adenomas are seen in minor salivary glands, amongst which palate is one of the site [1]. Other sites include submandibular gland (8%), lips, buccal mucosa, gingiva and tongue. It is composed of epithelial and myoepithelial cells arranged with various morphological patterns, demarcated from surrounding tissues by a fibrous capsule [3]. It also ranks first as the most common tumor of the intraoral salivary glands of which palate is the most common intraoral site, followed by upper lip and buccal mucosa. Muco-epidermoid carcinoma is the most common malignant salivary gland tumor, while pleomorphic adenoma is the most common benign counterpart. Pleomorphic adenoma of the palate is rare [3]. Patients with pleomorphic adenomas of the minor salivary glands present mostly in fourth to sixth decades, with a slight predominance in female [4]. They usually present as a unilateral, painless, slow-growing mass in the parotid gland. However, when they originate in the hard and soft palate they present typically as a firm or rubbery submucosal mass without ulceration or surrounding inflammation.

The differential diagnoses for this case include palatal abscess, odontogenic and non-odontogenic cysts, and other soft tissue tumors. Abscess can be ruled out because of loss of signs and symptoms of inflammation, whereas cysts are not firm in consistency. FNA biopsy should be performed as an adjunct to diagnosis prior to definitive surgical treatment. Computed tomography or magnetic resonance imaging should be considered when assessing for presence of bony erosion or soft tissue and nerve involvement [5]. The histological pictures of pleomorphic adenomas vary. Pleomorphic adenomas of the extramajor salivary glands are similar to those in the major salivary glands and are composed of a mixture of epithelial and stromal elements. Three main histologic subgroups have been identified: myxoid (80% stroma), cellular (myoepithelial predominant), and mixed (classic) type [6]. Surgical excision is the treatment of choice. Longevity and recurrence are risk factors for malignant transformation. The propensity for malignant transformation is documented to be 1.9-23.3% [7]. We performed complete excision of tumor with overlying mucosa and surgical wound was closed with advancement of adjacent mucosa. This produced an excellent result. The excised region can be left to heal by secondary intention also. In this case the tumor itself served as tissue expander, to advance adequate mucosal coverage, and achieve primary healing.

## Conclusion

Pleomorphic adenoma, though a common entity, is still a challenging tumor for pathologist, radiologist, and the surgeon. Its diverse histological and topographical property makes the tumor special. The examining clinician and treating surgeon must be aware of its recurrence, longevity, and malignant potential if incorrectly diagnosed or treated.
